# Consumption Patterns of Alcohol and Alcohol mixed with Energy Drinks in Australian Students and Non-Students

**DOI:** 10.3390/nu12010149

**Published:** 2020-01-05

**Authors:** Sarah Benson, Joris C. Verster, Andrew Scholey

**Affiliations:** 1 Centre for Human Psychopharmacology, Swinburne University, Melbourne, VIC 3122, Australia; 2 Division of Pharmacology, Utrecht Institute for Pharmaceutical Sciences, Utrecht University, 3584CG Utrecht, The Netherlands

**Keywords:** energy drink, alcohol, caffeine, AMED, alcohol consumption, consequences, student drinking

## Abstract

Studies assessing alcohol mixed with energy drink (AMED) use and drinking behaviors have been largely restricted to student-only cohorts. Thus, it is not known whether evidence from these studies is applicable to non-student populations. This study examined alcohol consumption and involvement in negative alcohol-related consequences among AMED and alcohol-only (AO) users, with the aim of determining whether drinking behaviors differ according to student status. An online survey was conducted in Australia to assess alcohol consumption and alcohol-related consequences following AMED and AO consumption, according to student status. The final sample consisted of 1369 participants. Between-subjects analyses comparing AMED and AO users, confirmed previous findings in that, compared with AO users, AMED users consumed significantly more alcohol, consumed alcohol more frequently and were involved in a greater number of alcohol-related consequences. Within-subjects analyses of AMED users comparing AMED and AO drinking occasions revealed that significantly less alcohol was consumed and involvement in negative alcohol-related consequences was lower during AMED compared with AO drinking occasions. Regardless of drink type, compared with students, non-students consumed more alcohol, consumed alcohol more frequently and were involved in a greater number of negative alcohol-related consequences. These findings provide further evidence that AMED use is one manifestation of a risk-taking personality and suggest that non-students drink more alcohol, drink more frequently and are involved in a greater number of negative alcohol-related consequences than students.

## 1. Introduction

Worldwide, over two billion people use alcohol and an estimated 283 million people have an alcohol use disorder [1]. Alcohol consumption is particularly problematic in Australia, which ranks alongside countries with the highest consumption levels worldwide [2]. This is largely driven by the high prevalence of young adults who ‘binge drink’—that is, consume >4 standard drinks per drinking occasion.

Binge drinking accounts for the bulk of alcohol-related morbidity among young adults [3] and is associated with many adverse outcomes such as physical injury, accidents, road trauma, alcohol poisoning, memory loss, self-harm, sexual risk-taking, aggression and violence [4,5,6,7,8,9]. Given the positive association between binge drinking and harms, young Australian adults are at a heightened risk of negative alcohol-related outcomes. 

Over the past decade, it has become increasingly popular for young adults to consume alcohol mixed with energy drink (AMED). Surveys generally report that 20%–25% of their samples had used AMED within the previous month [10,11,12]. AMED use has been consistently associated with increased alcohol consumption [10,11,13,14], possibly through ‘masking’ of subjective intoxication. Findings from recent meta-analyses, however, indicate that AMED consumption does not result in masking [15], nor does it directly increase alcohol consumption [16]. Using between-subjects analysis, Verster et al. [16] found that during typical drinking occasions and during the past month’s heaviest drinking occasion, AMED users consumed significantly more alcohol compared to alcohol-only (AO) users. However, within-subjects analyses, restricted to AMED users when consuming AMED vs. consuming AO, revealed that consumption does not differ between AMED and AO occasions. Furthermore, consumption during the past month’s heaviest drinking occasion was significantly less during the AMED compared to AO drinking occasion. These findings indicate that rather than AMED directly causing increased alcohol consumption, AMED use is one manifestation of an underlying trait associated with heavy drinking. 

The between-subjects comparison in the latter meta-analysis was based on 9 studies [16]—from which, all but three were restricted to student cohorts, (two Lubman et al. studies and Trapp et al.) [17,18]. Similarly, the within-subjects analysis included seven studies [16], with only three including non-students, (the two Lubman et al. studies from the between-subjects analysis and Peacock et al. [19]). While the effect of student status on alcohol consumption was not a primary focus of these studies, Lubman et al. [17] reported that the odds of consuming three AMEDs per drinking occasion were higher for students than those who worked full time. Unfortunately, the statistical significance of this finding is unknown, as only the odds ratio value was reported and the confidence intervals and p-values were not reported. Trapp et al. [18] found that being in part-time or full-time employment significantly increased the odds of being an energy drink user but did not assess the effects of employment on AMED use. The effects of student status were not reported by the second Lubman et al. study [17] and Peacock et al. [19]. Thus, despite the vast majority of studies assessing AMED use in the context of alcohol consumption using student-only samples, it is not known whether students are in fact a unique or key subgroup, or the extent to which results generalize to the rest of the population. The current study addresses this issue.

Our study is a partial replication of de Haan et al. [12] and Johnson et al. [20], who surveyed 6002 Dutch and 1873 UK university students, respectively. It expands on previous studies by assessing a community sample, including both students and non-students, of young Australians. Both de Haan et al. [12] and Johnson et al. [20] found AMED and alcohol consumption results consistent with those of Verster et al. [16]. Furthermore, findings of de Haan et al. [12] and Johnson et al. [20] support previous research [10,11,21] showing that AMED users are involved in a greater number of negative drinking consequences, for example, involvement in sexual situations that were later regretted and passing out from drinking, but AMED users partake in fewer negative consequences during AMED compared to AO drinking occasions. 

The primary aim of this study was to determine whether alcohol behaviors, in the context of AMED, differ according to student status. Our further aims were to assess alcohol consumption and involvement in negative alcohol-related consequences in i) AMED compared to AO users and ii) AMED users on AMED and AO drinking occasions. Due to the mixed results from previous research assessing AMED use and student status, we did not have a hypothesis regarding the direction of any difference based on student status, alcohol consumption and involvement in alcohol-related consequences. Rather the aim was to resolve the differences from previous studies in a large sample by directly comparing students and non-students. Consistent with previous research, we hypothesized that AMED users will consume more alcohol and be involved in a greater number of negative alcohol-related consequences compared to AO users. Furthermore, we predicted that AMED users will not consume more alcohol during AMED compared and AO drinking occasions and will partake in fewer negative alcohol-related consequences during AMED occasions. 

## 2. Materials and Methods 

This cross-sectional study was an online survey assessing alcohol consumption and involvement in negative alcohol-related consequences in i) AMED users compared to non-users ii) AMED users across AMED and alcohol-only (AO) drinking occasions and iii) students compared to non-students. The study was approved by the Swinburne University Human Research Ethics Committee (Reference 2012/045) and conducted in accordance with the Declaration of Helsinki.

### 2.1. Sample

Participants were recruited via word of mouth, advertisements on social media (i.e., Facebook) and flyers. Facebook advertising was targeted towards users in Australia and flyers were distributed by hand and placed on community boards. Participation was voluntary and anonymous, and participants were offered the opportunity to enter a prize draw at the end of the survey to win one of three iPads. It was a requirement that participants resided in Australia and were aged 18 years or older. Participants were asked their state/territory of residence and their age. Any participant who did not meet the inclusion criteria was excluded from further participation. In total 1748 people opened the survey link. After cleaning the data set for participants who answered ‘no’ to whether they had answered all questions honestly, and those who had opened the survey but provided unusable data, 1369 datasets remained. 

### 2.2. Survey Outline

Following the informed consent procedures, participants answered questions assessing demographics and use of tobacco and illicit drugs. Participants were then asked a series of questions assessing their consumption of alcohol on occasions when (i) alcohol was used without a mixer and (ii) alcohol was mixed with energy drink, see Table 1. Consistent with previous research [12,20], alcohol-only (AO) occasions were defined as consuming alcohol without a mixer (i.e., drinking beer, wine or straight spirits) and AMED occasions were defined as consuming energy drinks concomitantly or within two hours prior or post consuming alcohol. 

Alcohol consumption was defined using standardized Australian alcohol units (1 standard drink = 10 grams of pure alcohol) and one energy drink was defined as a standard 250 ml can, containing 80 mg caffeine. Participants were informed of these definitions and were shown pictures of various standard drinks and energy drink cans. The consumption questions were adapted from the Quick Drinking Screen (QDS) [22] and assessed frequency and quantity of alcohol consumed across various timescales (i.e., per occasion, 30 days and 12 months) for each drink type. The QDS contains 4 items measuring alcohol consumption and has been shown to be highly reliable and valid [23,24,25]. 

Negative alcohol-related consequences were measured using the Brief Young Adult Alcohol Consequences Questionnaire (BYAACQ) [26]. The BYAACQ contains 24 items assessing possible consequences of alcohol consumption, for example, “I have found it difficult to limit how much I drink” and “My drinking has got me into sexual situations I later regretted”. Participants were asked to indicate whether the statement was applicable to them within the previous year by responding “yes” or “no”. The total BYACCQ scores range from 0 to 24, with higher scores indicating greater involvement in negative consequences. Participants completed the BYAACQ for AO and AMED drinking occasions, providing they had participated in those drinking occasions. 

### 2.3. Data Collection and Statistical Analyses

Data were collected online using SurveyMonkey and analyzed using the Statistical Package for the Social Sciences version 24 (SPSS Inc., Chicago, IL, USA). All tests were two tailed and differences were regarded as significant at *p* < 0.05. The mean, standard deviation and frequency distribution were calculated for the alcohol consumption and BYAACQ scores for both AO and AMED occasions, and students and non-students. 

Between-subjects demographic comparisons were made using a series of independent samples t-tests for continuous variables and Chi Square tests for nominal variables. The AMED group consisted of any participant who had used AMED while the AO group consisted of any participant who had used alcohol without a mixer and never mixed with energy drink (i.e., consumed beer, wine, straight spirits). Student status was determined according to whether the participant was or was not a University student at the time of completing the survey. 

The alcohol consumption and BYAACQ score outcome variables were non-normally distributed. We therefore used Generalized Linear Modelling (GLM), specifically, the negative binomial (NB) distribution with log link, to assess between-subjects effects. NB is appropriate for positively skewed count data with overdispersion, i.e., having a variance which is greater than the mean. Positively skewed and overdispersed data is common in alcohol consumption data [27,28,29] whereby many participants consume a couple of drinks and a long positive tale of participants consume a higher number of drinks. 

To assess within-subjects effects in AMED users, Generalized Estimating Equations (GEE), the repeated measures equivalent of GLM, was used. A series of GEE, NB distribution with log link, were used to assess the effects of drink-type occasion (AMED versus AO) and student status (student versus non-student) on the drinking and BYAACQ score outcome variables. 

## 3. Results

### 3.1. Demographics

Between-group analyses revealed significant differences according to consumer type and student status. AMED users were significantly more likely to be male, younger, use illicit drugs and smoke, and first and regularly use alcohol at a younger age than AO users. These results remained consistent in the student-only sample and only gender was not significantly different in the non-student sample. Students were more likely to be female, younger and non-smokers, compared to non-students, see Table 2.

### 3.2. Between-Subjects Comparisons

As shown in Table 3, there were significant effects of consumer type and student status on each of the drinking outcome variables and the BYAACQ score. AMED users and non-students consumed significantly more alcohol, consumed alcohol more frequently and participated in a greater number of negative alcohol-related consequences compared to AO users and students, respectively. We did not find any consumer type*student status interactions.

### 3.3. Within-Subjects Comparisons of the AMED Group According to Drink-Type Occasion and Student Status

As shown in Table 4, each alcohol consumption item and the BYAACQ score differed significantly according to drink-type occasion. AMED users consumed significantly more alcohol, consumed alcohol more frequently and were involved in more negative alcohol-related consequences during AO compared to AMED drinking occasions. Non-students participated in a significantly greater number of days spent drinking, binge drinking and getting drunk in the past month, consumed a greater number of drinks in the past year and were involvement in more negative alcohol-related consequences compared to students. Significant drink-type occasion*student status ordinal interactions were found on the average number of drinks consumed per drinking occasion (β = 0.20, β SE = 0.07, IRR = 1.22, 95% CI = 1.07–1.40, *p* = 0.004) and the number of days drunk within the previous month (β = −0.30, β SE = 0.15, IRR = 0.75, 95% CI = 0.56–0.10, *p* = 0.048).

## 4. Discussion

This study aimed to determine whether AMED use results in increased alcohol consumption and involvement in negative alcohol-related behaviors, and whether drinking behaviors differ according to student status. Our results confirm that AMED users consume more alcohol, drink alcohol more frequently, and partake in more negative alcohol-related behaviors than AO users. However, on drinking occasions when AMED users mix alcohol with energy drink, they consume less alcohol and are involved in fewer negative alcohol-related consequences compared to occasions when AO is consumed. Comparisons according to student status revealed that non-students consumed more alcohol, consumed alcohol more frequently and were involved in more negative alcohol-related consequences compared to students, and this pattern remained consistent in the AMED-user subgroup. These findings support the notion that AMED use is partly a manifestation of a high-risk ‘phenotype’ [16]. Further support for this notion comes from comparison of other demographic features between AMED and AO cohorts. These demonstrate that in addition to their AMED use, individuals in this group are more likely to smoke, use illicit drugs, to use alcohol for the first time and begin drinking regularly at a younger age compared to AO users. 

Our findings suggest that non-students form an important demographic who should not be overlooked in AMED research. Regardless of drink-type occasion, non-students consumed more alcohol, drank more frequently and were involved in more negative alcohol-related consequences compared to students. Within AMED users, the general pattern of decreased alcohol consumption and frequency of intoxication during AMED compared to AO drinking occasions was confirmed in non-students. In fact, the differences between AMED and AO occasions were more marked for non-students than for students. That is, the association between AMED occasion and reduced alcohol intake, as well as fewer AMED drinking occasions, was greater in non-students. Our findings contradict those of Lubman et al. [17], the only known previous study assessing the effect of AMED with respect to student status, who found that, compared with those who work full time, students were more likely to consume three AMEDs per drinking occasion. However, as p-values were not reported, it is not known whether this difference was statistically significant. Furthermore, as Lubman et al. [17] assessed AMED use at a level of three AMEDs per occasion and we assessed maximum intake, it may be that student status differentially affects AMED use in relation to quantity consumed. Both the current study and Lubman’s studies Australian cohorts. In Australia, AMEDs are commonly double the price of many AO beverages and this may be a stronger deterrent for students than for non-students to restrict the number of AMED purchases. Unfortunately, we did not collect any data assessing salary to test this. Overall, our findings may be of clinical importance as they indicate that AMED use may be an indicator of problematic behaviors, such as excessive drinking and risk-taking. Thus, AMED use may be amongst indicators used to identify individuals who may benefit from risk and harm reduction strategies. 

The results of this study should be considered in light of several methodological limitations. The external validity of our results is restricted to an Australian community sample and although the general AO and AMED drinking patterns found were consistent with those of studies in other locations, it may be that AMED has a differential effect according to student status among various cultures. Furthermore, as students and non-students were not matched, it is possible that other group differences may have affected the findings. Secondly, as with other surveys, this study relied on participant recall, which can be problematic, particularly when memory lapse is a symptom of intoxication and participants were asked to recall drinks consumed one and twelve months prior. Previous research has demonstrated that recall accuracy is increasingly susceptible to bias over time [30,31,32] and that heavy drinking is associated with under-reporting alcohol consumption while light drinking is associated with over-reporting alcohol intake [33,34]. Furthermore, it is possible that recall accuracy is differentially affected according to demographic traits, for example, gender, student status or age. However, by using a within-subjects design, any between-subjects differences in recall bias are minimized. Lastly, as participation in AMED drinking was less frequent than AO drinking, this may have differentially affected recall for AO and AMED events. As our study was the first to assess statistical differences in AMED and AO use, and resulting involvement in negative alcohol-related consequences according to student status, additional research is needed to replicate these findings in other samples. Future AMED research should aim to include non-students in their samples and determine whether drinking outcomes following AMED use are specific to energy drink use only or are broadly associated with caffeine intake and as such, can be applied to mixing alcohol with other caffeinated beverages such as coffee and cola beverages.

## 5. Conclusions

In conclusion, this study provides further evidence that AMED use is one of several manifestations of a risk-taking phenotype or trait. The results also suggest that non-students drink more alcohol, drink more frequently and are involved in a greater number of negative alcohol-related consequences than students, regardless of whether AMED or AO is consumed. This new finding needs to be replicated both in Australia and in other jurisdictions. Clinicians and other health care workers should be aware of the risk-taking behaviors associated with AMED use, which could prove useful in identifying individuals at high risk of excessive drinking and associated harms.

## Figures and Tables

**Table 1:** Alcohol consumption questions.

**AO**
How many standard alcoholic drinks do you usually have on one occasion?
In the past 30 days, how many days did you drink alcohol?
In the past 30 days, how many days did you get drunk?
In the past 30 days, how many times did you have >5 (male)/4 (female) alcohol drinks on one occasion?
In the past 30 days, what is the greatest number of alcoholic drinks you had on one occasion?
On that occasion (previous question), how many hours did you consume alcohol?
In the past 12 months, what was the greatest number of alcoholic drinks you consumed on one occasion?
**AMED**
When you combine, how many standard alcoholic drinks do you usually have on one occasion?
In the past 30 days, how many days did you combine energy drinks and alcohol?
In the past 30 days, while combining, how many days did you get drunk?
While combining in the past 30 days, how many times did you have >5 (male)/4 (female) alcohol drinks on one occasion?
While combining in the past 30 days, what is the greatest number of alcoholic drinks you had on one occasion?
On that occasion (previous question), how many hours did you consume alcohol?
While combing in the past 12 months, what was the greatest number of alcoholic drinks you consumed on one occasion?

Note: AO: alcohol only; AMED: alcohol mixed with energy drink.

**Table 2:** Sample characteristics by student status and AMED/AO use. Except for %, numbers are means (with SD in parentheses).

	Consumer Type			Student Status			Consumer Type by Student Status							
	AMED (*n* = 417)	AO (*n* = 952)	*d*	Student (*n* = 863)	Non-Student (*n* = 506)	*d*	Student			Non-Student			Student vs. Non-Student	
													AMED	AO
							AMED (*n* = 234)	AO (*n* = 629)	*d*	AMED (*n* = 183)	AO (*n* = 323)	*d*	*d*	*d*
Male/Female(%)	44.84/ 55.16	34.77/ 65.23	0.19 **	30.71/ 69.29	50.00/ 50.00	0.39 **	36.32/ 63.68	28.62/ 71.38	0.15 *	55.74/ 44.26	46.75/ 53.25	0.17	0.40 **	0.37 **
Age (years)	22.82 (4.57)	24.47 (6.00)	0.29 **	23.13 (5.52)	25.40 (5.59)	0.41 **	22.02 (4.24)	23.54 (5.88)	0.28 **	23.86 (4.78)	26.27 (5.83)	0.44 **	0.41 **	0.47 **
Illicit drug use (past year, %)	33.81	20.48	0.29 **	23.29	26.68	0.08	34.19	19.24	0.32 **	33.33	22.91	0.23 *	0.03	0.09
Current smoker (%)	20.38	12.61	0.20 **	12.17	19.76	0.21 **	15.81	10.81	0.14 *	26.23	16.10	0.25 *	0.26 *	0.15 *
Age first used alcohol (years)	14.36 (2.68)	15.03 (2.91)	0.24 **	14.94 (2.84)	14.64 (2.87)	0.11	14.59 (2.65)	15.07 (2.90)	0.17 *	14.07 (2.71)	14.96 (2.91)	0.31 *	0.19	0.04
Age used alcohol regularly (years)	17.32 (2.03)	18.22 (2.51)	0.38 **	17.92 (2.36)	17.98 (2.48)	0.02	17.37 (2.01)	18.13 (2.45)	0.33 **	17.26 (2.05)	18.39 (2.61)	0.47 **	0.05	0.10

Note: * *p* < 0.05, ** *p* < 0.001; AO: Alcohol only; AMED: Alcohol mixed with energy drink; CI: confidence interval; $\bar{x}$(SD): mean and standard deviation; *n*: number of participants, *d*: Cohen’s d.

**Table 3:** Between-subjects comparisons according to consumer type and student status on occasions when alcohol-only was consumed.

	Means (Standard Deviations)								GLM Results							
	Consumer		Student Status		Student		Non-Student		Consumer Type				Student Status			
	AMED	AO	Student	Non-Student	AMED	AO	AMED	AO	β	β SE	IRR	95% CI	β	β SE	IRR	95% CI
Drinks/occasion	7.57 (4.45)	4.89 (3.81)	5.14 (3.55)	6.68 (4.96)	6.39 (3.76)	4.56 (3.28)	8.66 (4.94)	5.71 (4.59)	0.44	0.06	1.55 **	1.37–1.75	0.26	0.06	1.30 **	1.15–1.46
Drinking days/month	8.26 (6.98)	6.49 (6.49)	6.17 (5.97)	8.49 (7.55)	7.21 (6.20)	5.69 (5.82)	9.42 (7.81)	7.97 (7.27)	0.24	0.06	1.27 **	1.13–1.44	0.32	0.06	1.38 **	1.23–1.55
Days drunk/month	3.79 (4.20)	2.17 (3.43)	2.41 (3.30)	3.10 (4.39)	3.37 (3.97)	1.98 (2.91)	4.18 (4.55)	2.58 (4.14)	0.56	0.07	1.74 **	1.53–1.99	0.26	0.07	1.29 **	1.14–1.47
Binge drinking/month	4.71 (4.56)	2.77 (3.85)	2.99 (3.86)	4.00 (4.61)	4.07 (4.12)	2.49 (3.64)	5.36 (5.09)	3.35 (4.13)	0.53	0.07	1.70 **	1.49–1.93	0.29	0.06	1.34 **	1.18–1.52
Greatest drinks/month	10.75 (6.85)	7.22 (5.91)	7.50 (5.82)	9.66 (7.13)	9.18 (5.75)	6.65 (5.52)	12.18 (7.63)	8.55 (6.61)	0.40	0.06	1.49 **	1.32–1.68	0.25	0.06	1.29 **	1.15–1.45
Drinking hours/month	6.22 (3.67)	4.78 (3.03)	4.91 (2.89)	5.75 (3.85)	5.70 (2.98)	4.56 (2.82)	6.74 (4.35)	5.25 (3.35)	0.26	0.06	1.30 **	1.15–1.47	0.16	0.06	1.17 *	1.04–1.32
Greatest drinks/year	16.11 (8.45)	11.40 (7.88)	11.58 (7.44)	14.96 (9.32)	14.15 (7.44)	10.40 (7.10)	18.11 (9.19)	13.38 (8.87)	0.35	0.06	1.41 **	1.26–1.59	0.26	0.06	1.29 **	1.15–1.45
BYAACQ Score	10.21 (5.45)	6.70 (4.80)	7.24 (4.88)	8.51 (5.73)	9.34 (4.81)	6.55 (4.70)	11.34 (6.02)	7.03 (4.97)	0.42	0.07	1.52 **	1.32–1.75	0.16	0.07	1.18 *	1.03–1.35

Note: ** *p* < 0.001; * *p* < 0.05; GLM: Generalized Linear Modelling; AMED: alcohol mixed with energy drink; AO: alcohol-only; IRR: incidence rate ratio.

**Table 4:** Within-subjects comparisons of the AMED group according to drink-type occasion and student status.

	Means (Standard Deviations)								GEE Results							
	Occasion		Student Status		AMED Occasion		AO Occasion		Drink-Type Occasion				Student Status			
	AMED	AO	Student	Non-Student	Student	Non-Student	Student	Non-Student	β	Β SE	IRR	95% CI	β	β SE	IRR	95% CI
Drinks/occasion	5.45 (4.13)	6.88 (4.52)	5.51 (3.69)	7.08 (5.04)	5.14 (3.66)	5.87 (4.68)	5.86 (3.70)	8.18 (5.12)	0.23	0.03	1.26 **	1.18–1.35	0.25	0.06	1.29 **	1.14–1.45
Drinking days/month	1.90 (3.05)	8.18 (6.95)	4.47 (5.43)	6.05 (7.13)	1.54 (2.09)	2.39 (3.95)	7.25 (6.13)	9.36 (7.74)	1.46	0.08	4.29 **	3.69–4.99	0.30	0.08	1.36 **	1.16–1.59
Days drunk/month	1.53 (2.72)	3.64 (4.05)	2.29 (3.19)	3.06 (4.10)	1.21 (1.82)	1.97 (3.56)	3.32 (3.82)	4.04 (4.31)	0.87	0.08	2.38 **	2.04–2.77	0.29	0.11	1.34 *	1.07–1.67
Binge drinking/month	1.61 (2.61)	4.55 (4.45)	2.73 (3.48)	3.66 (4.74)	1.33 (1.99)	1.99 (3.24)	4.07 (4.03)	5.16 (4.89)	1.04	0.08	2.83 **	2.44–3.28	0.29	0.10	1.34 *	1.11–1.62
Greatest drinks/month	5.74 (6.38)	10.49 (6.73)	7.42 (6.33)	9.20 (7.64)	5.38 (6.23)	6.22 (6.55)	9.37 (5.79)	11.93 (7.56)	0.60	0.05	1.83 **	1.66–2.00	0.22	0.07	1.24 *	1.08–1.42
Drinking hours/month	4.47 (3.65)	5.95 (3.57)	4.85 (3.13)	5.73 (4.25)	4.20 (3.36)	4.84 (3.98	5.49 (2.74)	6.54 (4.34)	0.29	0.04	1.31 **	1.24–1.43	0.17	0.06	1.18 *	1.05–1.33
Greatest drinks/year	5.86 (6.13)	15.81 (8.35)	9.91 (8.09)	12.47 (9.65)	5.28 (5.79)	6.64 (6.49)	14.32 (7.48)	17.73 (9.01)	0.99	0.05	2.70 **	2.45–2.98	0.23	0.05	1.26 **	1.13–1.40
BYAACQ Score	7.15 (5.30)	9.63 (5.44)	7.61 (4.77)	9.62 (6.21)	6.33 (4.36)	8.25 (6.20)	8.73 (4.84)	10.89 (5.98)	0.30	0.03	1.35 **	1.26–1.44	0.23	0.06	1.26 *	1.12–1.43

Notes: ** *p* < 0.001; * *p* < 0.05; GEE: Generalized Estimating Equations; AMED: alcohol mixed with energy drink; AO: alcohol-only; IRR: incidence rate ratio.
